# Central Venous Catheter-Related Tachycardia in the Newborn: Case Report and Literature Review

**DOI:** 10.1155/2016/6206358

**Published:** 2016-12-12

**Authors:** Aya Amer, Roland S. Broadbent, Liza Edmonds, Benjamin J. Wheeler

**Affiliations:** Department of Women's and Children's Health, University of Otago, Dunedin School of Medicine, P.O. Box 56, Dunedin 9054, New Zealand

## Abstract

Central venous access is an important aspect of neonatal intensive care management. Malpositioned central catheters have been reported to induce cardiac tachyarrhythmia in adult populations and there are case reports within the neonatal population. We present a case of a preterm neonate with a preexisting umbilical venous catheter (UVC), who then developed a supraventricular tachycardia (SVT). This was initially treated with intravenous adenosine with transient reversion. Catheter migration was subsequently detected, with the UVC tip located within the heart. Upon withdrawal of the UVC and a final dose of adenosine, the arrhythmia permanently resolved. Our literature review confirms that tachyarrhythmia is a rare but recognised neonatal complication of malpositioned central venous catheters. We recommend the immediate investigation of central catheter position when managing neonatal tachyarrhythmia, as catheter repositioning is an essential aspect of management.

## 1. Introduction

Obtaining central venous access, often via an umbilical venous catheter (UVC), is a common aspect of modern NICU care and allows for the reliable administration of fluid, nutrition, and essential medications to unwell neonates. Despite these benefits, complications can occur, often related to malposition, extravasation, and/or thrombosis [1, 2]. Resultant cardiac complications are rare but potentially life threatening [2]. In contrast, tachyarrhythmias, such as supraventricular tachycardia (SVT), are relatively common in neonates [3], but their occurrence as a complication of central venous access, particularly in association with prematurity, is not often seen or considered.

We present a case of SVT secondary to UVC migration and malposition in a 29-week gestation neonate and review the available literature. We conclude with a suggested approach to the evaluation, management, and prevention of neonatal tachyarrhythmias occurring in the context of central venous access.

## 2. Case Report

**Figure 1:**
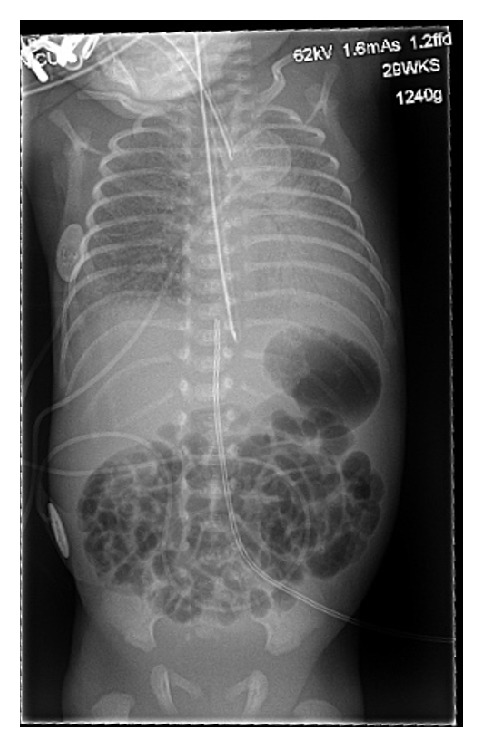
Initial postinsertion CXR, showing appropriate UVC placement, just below the diaphragm at T9.

A 1240 g (50th centile), 29-week gestation male twin (twin 2) was born following a pregnancy complicated by twin-to-twin transfusion (recipient twin). Delivery was via emergency lower segment caesarean section, due to foetal distress. Apgar scores at birth were 2 (1 minute), 6 (5 minutes), and 8 (10 minutes). Initial resuscitation included intubation and ventilation, as well as cardiac compressions for the first 2 minutes because of a sustained heart rate of less than 60 beats/minute. His subsequent management included surfactant administration, empiric antibiotics, caffeine, and conventional mechanical ventilation for 12 hours before being extubated onto Continuous Positive Airway Pressure (CPAP). In addition, as a routine part of NICU care, a 3.5 French double lumen UVC was inserted to a length of 7 cm. It was noted to be bleeding back easily, and an anterior-posterior (AP) chest X-ray (CXR) confirmed the catheter tip in a satisfactory position at T9, just below the diaphragm; see Figure 1.

**Figure 2:**
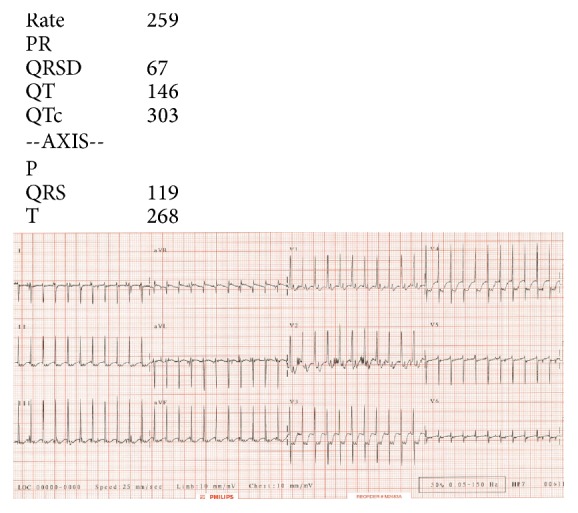
ECG demonstrating narrow complex tachycardia; rate: 260 beats/minute.

At 30 hours of age, he developed sudden onset tachycardia, with a heart rate of 250–270 beats/minute. He remained well perfused, with no respiratory or haemodynamic compromise. Mean arterial blood pressure (MABP) was 31 mmHg. An electrocardiogram (ECG) was diagnostic for SVT, demonstrating a narrow complex tachycardia with a rate of 259 beats/minute, a normal QTc of 303 ms, absent p-waves, and no flutter waves; see Figure 2.

**Figure 3:**
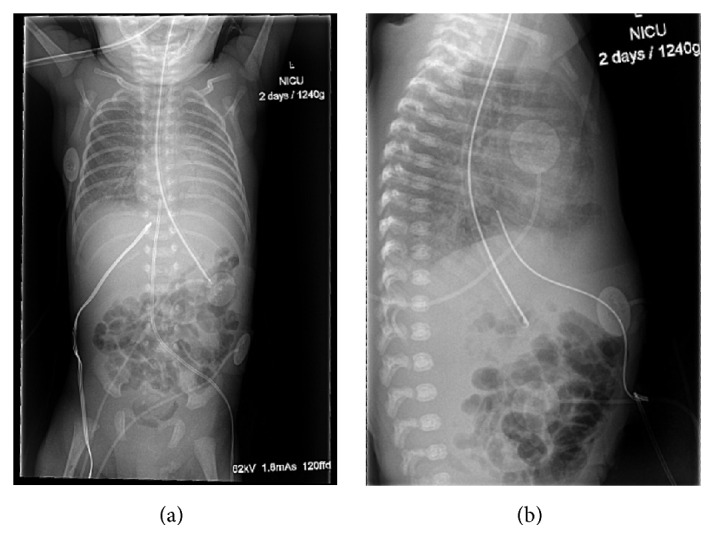
AP and lateral chest and abdomen X-rays taken after onset of SVT. The tip of the catheter is seen to have migrated into the right atrium.

Vagal manoeuvres were ineffective. Adenosine was then administered starting with a dose of 50 mcg/kg, followed by 100 mcg/kg. Following a third dose (150 mcg/kg) he reverted to sinus rhythm. However, 20 minutes later SVT returned. This sequence (recurrent SVT and then nonsustained response to adenosine) repeated itself twice over the next 45 minutes with subsequent doses of adenosine, given at incrementally increasing doses of 50 mcg/kg to a maximum of 300 mcg/kg. While this was occurring, AP and lateral CXRs were performed to assess the UVC position. These demonstrated migration of the UVC tip into the right atrium; see Figure 3.

The UVC was pulled back 1 cm under aseptic technique. At this point a final dose of adenosine (300 mcg/kg) was given which resulted in permanent reversion to sinus rhythm.

Throughout these events the infant had no evidence of cardiovascular compromise. His MABP remained stable at 35 mmHg. He however did require a small temporary increase in his oxygen requirement. After 11 weeks he was discharged home without any cardiovascular concerns or sequelae.

## 3. Discussion

We have described the rare scenario of SVT as a consequence of UVC migration and malposition. While tachyarrhythmias are well recognised as a potential complication of central venous catheters in adults, only scattered case reports exist in the neonatal literature [3]. 16 cases of atrial tachyarrhythmia associated with central venous access in neonates are available to review when combined with our case [1, 3–12]. These are presented in Table 1. Atrial flutter (8/16) and SVT (7/16) are the two common rhythms described. An awareness of this pattern is vital, as atrial flutter in a non-catheter-related context is less common than SVT and thus potentially prone to underrecognition [3]. Promptly and accurately distinguishing the type of arrhythmia is particularly important as treatment differs, with synchronised cardioversion for atrial flutter versus intravenous adenosine for SVT [1, 3–10, 12].

Migration of central venous catheters in neonates is well known in clinical practice but has not been well studied. Both Peripheral Inserted Central Catheters (PICCs) and UVCs are implicated, with a recent case series reporting migration in up to 23% of UVCs at 24 hours [13]. This migration may occur for a number of reasons, including contraction of the umbilical stump and changes in size of the abdomen (in the case of UVCs); recurrent movement of the affected limb or head; and routine flushing and handling of the catheter by nursing/medical staff. Therefore, correct initial positioning of the catheter tip upon insertion does not preclude the central line as a cause for a subsequent arrhythmia and serial imaging should be considered as a way of confirming catheter tip location. The validation of ultrasound for localisation of catheters tips is a welcome advance [1]. Understanding the potential for catheter migration and subsequent arrhythmia is also important as time to onset varies. While the majority occur at the time of insertion, arrhythmia can occur hours or even days after insertion (in one case, 47 days after insertion) [6, 9, 11, 12]. This is demonstrated by our case, with migration of the catheter tip implicated in SVT onset more than a day after insertion.

There are several proposed mechanisms for arrhythmia induction. It could be that there are premature atrial beats induced when the catheter tip comes into contact with the endocardium, thus triggering an SVT in the presence of an accessory pathway [1]. Another possible mechanism is that the catheter could cause mechanical distortion of the atria, predisposing to the development of a reentry pathway [3].

Either way, in the majority of cases catheter tip withdrawal appears important but alone may not induce reversion to sinus rhythm, with medical therapy also usually required. In all cases reported, once the catheter was removed or withdrawn to a satisfactory position ± definitive medical therapy, there were no further recurrences of arrhythmia.

In conclusion, atrial tachyarrhythmia must be added to the range of dangerous complications of central indwelling catheters in the neonates. This report is intended to highlight this complication, raise awareness, and provide a more complete description of this rare adverse event. Confirmation of arrhythmia type (SVT versus atrial flutter) and determining catheter position are critical aspects of acute management. Withdrawal of the catheter to sit outside the heart should occur before cardioversion. This case is also a salient reminder that UVCs can migrate following their initial placement, and consideration should be given to serial catheter imaging as part of a program aimed at reducing catheter-related complications.

## Figures and Tables

**Table 1:** Reported cases of rapid atrial arrhythmias associated with central venous catheters in neonates.

Author (year)	Cases (*n*)	Catheter type	Catheter position	Interval between insertion and onset of arrhythmia	Arrhythmia type	Treatment
Dunnigan et al. (1985)	3	UVC	Right atrium	Day of insertion (time not recorded)	Atrial flutter ×3	Transoesophageal pacing
Leroy et al. (2002)	1	UVC	Left atrium	Time of insertion	Atrial flutter	Transoesophageal pacing
Sinha et al. (2005)	1	UVC	5th thoracic vertebra	Immediate	Atrial flutter	Synchronised cardioversion
Verheij et al. (2009)	2	UVC	6th thoracic vertebra 7th thoracic vertebra	Time of insertion ×2	SVT Atrial flutter	Adenosine^*∗*^ Synchronised cardioversion^*∗*^
de Almeida et al. (2016)	1	UVC	Left atrium	12 hours	SVT	Synchronised cardioversion
Current case: Amer et al. (2016)	1	UVC	Right atrium	30 hours	SVT	Adenosine^*∗*^ Catheter withdrawal
Obidi et al. (2006)	1	PICC	Right atrium	48 hours	Atrial flutter	Synchronised cardioversion
Thyoka et al. (2014)	1	PICC	Right atrium	Day of insertion	SVT	Adenosine
Daniels et al. (1984)	2	External jugular	Right atrium	Time of insertion Day 47	SVT Atrial flutter	Synchronised cardioversion^*∗*^
Da Silva and Waisberg (2010)	1	External jugular	Mid SVC (withdrawn 1 cm)	Time of insertion	SVT	Synchronised cardioversion
Conwell et al. (1993)	1	Right femoral	Right atrium	48 hours	Ectopic atrial tachycardia	Catheter withdrawn
Casta et al. (2008)	1	Internal jugular^‡^	Mid SVC^‡^	Time of insertion	SVT	Adenosine Synchronised cardioversion

*∗*: arrhythmia recurred prior to catheter tip being sufficiently withdrawn.

‡: arrhythmia thought to be due to transoesophageal echo probe, though internal jugular and femoral venous line were also present at this time.
